# Analyses on the Temporal and Spatial Characteristics of Water Quality in a Seagoing River Using Multivariate Statistical Techniques: A Case Study in the Duliujian River, China

**DOI:** 10.3390/ijerph16061020

**Published:** 2019-03-20

**Authors:** Xuewei Sun, Huayong Zhang, Meifang Zhong, Zhongyu Wang, Xiaoqian Liang, Tousheng Huang, Hai Huang

**Affiliations:** Research Center for Engineering Ecology and Nonlinear Science, North China Electric Power University, Beijing 102206, China; xuewei_sun@ncepu.edu.cn (X.S.); 1172111022@ncepu.edu.cn (M.Z.); zhy_wang@ncepu.edu.cn (Z.W.); 1162211050@ncepu.edu.cn (X.L.); 50902253@ncepu.edu.cn (T.H.); huanghai@ncepu.edu.cn (H.H.)

**Keywords:** river water quality, multivariate statistical analysis, seagoing river, Duliujian River, environmental management

## Abstract

In the Duliujian River, 12 water environmental parameters corresponding to 45 sampling sites were analyzed over four seasons. With a statistics test (Spearman correlation coefficient) and multivariate statistical methods, including cluster analysis (CA) and principal components analysis (PCA), the river water quality temporal and spatial patterns were analyzed to evaluate the pollution status and identify the potential pollution sources along the river. CA and PCA results on spatial scale revealed that the upstream was slightly polluted by domestic sewage, while the upper-middle reach was highly polluted due to the sewage from feed mills, furniture and pharmaceutical factories. The middle-lower reach, moderately polluted by sewage from textile, pharmaceutical, petroleum and oil refinery factories as well as fisheries and livestock activities, demonstrated the water purification role of wetland reserves. Seawater intrusion caused serious water pollution in the estuary. Through temporal CA, the four seasons were grouped into three clusters consistent with the hydrological mean, high and low flow periods. The temporal PCA results suggested that nutrient control was the primary task in mean flow period and the monitoring of effluents from feed mills, petrochemical and pharmaceutical factories is more important in the high flow period, while the wastewater from domestic and livestock should be monitored carefully in low flow periods. The results may provide some guidance or inspiration for environmental management.

## 1. Introduction

River ecosystems have received more attention in recent years because they not only provide water resources for socioeconomic development, but they also play an important role in ecological integrity. The surface water quality in rivers plays an important role in the productivity and life of surrounding human societies. However, water quality deterioration in rivers has been extremely serious in developing countries due to the intensive anthropogenic activities along the river and lack of water treatment in the entire basin [1,2,3]. Nowadays, abundant literature focusing on the water quality in inland rivers or coastal seas can be easily found, while there is little reported information on seagoing rivers due to the complexity of their ecosystems and diversity of pollution sources [2,4]. Due to the transition from fresh water to sea water, seagoing rivers not only have the pollution characteristics of inland rivers, but also receive marine pollution through seawater intrusion. Due to their enormous economic value and various ecological functions, more attention should be paid to spatial and temporal variations of water quality in seagoing rivers.

Long-term water quality monitoring is the most common and effective method to evaluate eutrophication and other environmental problems since the spatial and temporal variations of these physicochemical parameters and biological indicators can be presented clearly and can further help researchers to evaluate the pollution status [5]. Generally, dissolved oxygen (DO), total nitrogen (TN), total phosphorus (TP) and dissolved inorganic nitrogen (DIN) are monitored as physicochemical indicators of river water quality [6,7,8]. Nitrogen pollution, which is related to land use change, soil nitrogen derived from agriculture, nitrification and denitrification [9,10], is the predominant factor attributed to the eutrophication problems in water [11,12]. Excessive phosphorus, which stems from fertilizers, feed, industrial waste and internal sediment release is another leading factor in the deterioration of water ecosystems [6,7,13,14]. Chlorophyll a (Chla), a biological indicator, reflects the primary production in aquatic ecosystems [8,15,16]. For a seagoing river, electrical conductivity (EC) and total dissolved solids (TDS) should be monitored as water salinization indicators reflecting the influence of seawater intrusion and soil erosion [17,18]. In two studies in Turkey, the pH in inland rivers (7.88–8.94) was much more stable than in coastal areas (6.53–9.91), and may therefore have an impact on fresh water during the process of seawater intrusion [18,19]. However, seawater intrusion complicates the quantitative analyses of the river water quality with simple statistical methods. As such, further analyses on the long-term water quality monitoring data based on more powerful analysis methods, which may identify the pollution source contributions more clearly, has become much more important for managers to implement better treatment measures [20,21].

Spearman correlation coefficient (a statistical test) and multivariate statistical methods, including cluster analysis (CA) and principal components analysis (PCA) are widely used to assess the seasonal and spatial variations of the water quality in rivers and coastal areas [22,23,24,25,26,27]. Many studies have concluded that natural processes like bedrock weathering, mineral oxidation, buffering processes, climatic conditions, soil erosion and seawater intrusion also have considerable effects on water quality [17,20,28]. In addition, the water quality degeneration is contributed to by anthropogenic activities such as agricultural fertilization and irrigation, domestic wastewater, livestock operation, over pumping, industrial and municipal effluents [17,22,29]. For coastal areas, water quality is polluted by excessive organic and inorganic loads, irregular opening of lagoons, as well as municipal wastewater and fish-farming [5,15,23]. Multivariate statistical analyses can mine potential relationships among water quality parameters, identify the main sources of pollution, and group the sampling sites into different clusters without losing important information [30,31,32].

Multiple water environmental parameters continuously measured in a natural seagoing river were analyzed to identify the spatial and temporal variation characteristics of water quality for seagoing rivers. The objective of this study is to find out the main pollutants in different seasons and river reaches by multivariate statistical methods, which are expected to help managers to understand the main pollution sources along the river and implement better treatment strategies.

## 2. Materials and Methods

### 2.1. Study Area

The Duliujian River, a seagoing river within the Haihe River Basin, located in southern Tianjin City, and flows from the conjunction of the Daqing River and the Ziya River to the Bohai Bay. With a total length of 70.14 km and a maximum width of 1 km in the upper and middle reaches, the Duliujian River has a catchment area of 3737 km^2^. The river basin belongs to the warm temperate zone with a semi-humid continental monsoon climate. The annual mean rainfall in this region is about 571 mm, but the rainfall is mainly concentrated from June to September. The monthly mean temperature ranges from −3.4 °C in January to 26.8 °C in July, while the average annual sunshine duration and evaporation are 2471 h and 1732 mm, respectively.

As the main water source supplying two important coastal wetland ecological environment conservation areas, namely the Beidagang Wetland Nature Reserve and the Tuanbo Birds and Nature reserve, the Duliujian River plays an important role in ecological environment protection. However, the Duliujian River also receives a large number of point and non-point sources pollutants throughout the year since that the up-middle reaches goes through the urban population living quarters and the downstream reach crosses some industrial parks that contain many textile, chemical, pharmaceutical, petroleum chemical, oil refinery, iron-steel and electroplating factories [20,28]. Furthermore, the wastewater from the aquaculture along the river and the surrounding areas also aggravates the pollution status of the Duliujian River.

### 2.2. Sampling and Analysis

**Figure 1:**
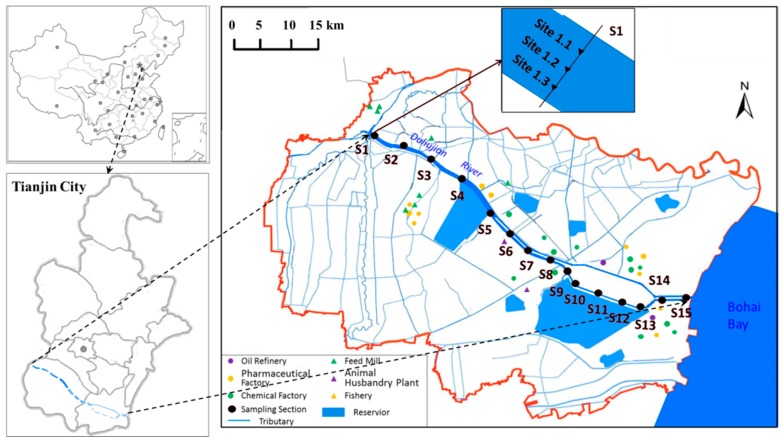
The sampling sections and sites in the study area.

In order to monitor the temporal and spatial changes of the water quality in the Duliujian River, 15 sampling cross-sections (S1–S15) were set along the whole Duliujian River considering natural conditions and human activities (Figure 1). Further, three sampling sites on each sampling cross-section were determined at 1/4, 1/2 and 3/4 across the river width. However, it should be stated that the sites S1.1–S2.3 on S1 and S2 in spring (May 2017) were not available due to dry up. Therefore, seasonal water samples from 45 sampling sites were collected in winter (December 2016, based on S1–S15), summer (July 2017, based on S1–S15) and autumn (September 2017, based on S1–S15), and 39 in spring (May 2017, based on S3–S15), respectively. During each sampling period, the work was carried out from S1 to S15 in sequence. In addition, weather forecasts for at least 15 days were checked before each sampling, and then seasonal sampling was completed in the consecutive days that were most suitable for sampling, in order to reduce the measurement errors caused by weather. At each sampling site, some water quality parameters were measured in situ like water temperature (WT), water depth (WD), and others, and at the same time, 1.5 L water was collected 1.0 m (1/2 of WD when WD < 1 m) below the water surface using a polymethylmethacrylate sampler and stored in a polyethylene plastic bottle. These sampling bottles were precleaned with detergent, rinsed three times with deionized water and dried under 30 °C in an oven for 24 h. Then, the water samples were placed in insulated boxes containing ice bags and transported to the laboratory for further analyses. The preservation and handling of the samples were all performed according to the Water Quality Sampling—Technical Regulation of the Preservation and Handing of Samples (HJ493-2009) norms [33]. Due to the normally closed sluices along the river, the average flow velocity was very slow. As such, in comparison with the nearest distance between two adjacent sections (3.0 km), the effects of pollutant transfer with water flow should be very slight and therefore ignored.

In Table 1, the monitored water quality parameters and their abbreviations, analytical methods and units are summarized. All parameters were measured or analyzed in accordance with relevant standard methods [34]. After filtering through 0.7 μm glass fiber filter and extraction for 12 h using 90% (*v*/*v*) acetone by a freeze-thaw method, the absorbance of the water samples was measured using a UV spectrophotometer, by which, the concentration of Chla could be calculated according to a relevant standard [35]. NH_4_^+^-N, NO_3_^−^-N, NO_2_^−^-N and PO_4_^3−^-P were determined from adequate samples filtered through 0.45 μm cellulose ester membranes. DIN was employed to represent the concentration of inorganic nitrogen and it was calculated using Equation (1):

DIN = NH_4_^+^-N + NO_3_^−^-N + NO_2_^−^-N. (1)

In order to ensure the quality of the experimental data, quality control procedures were performed throughout the sampling and analysis processes. In addition, two replicates of the parameters tested in laboratory for each sampling site were measured to minimize the experimental error and enhance the reliability of the measured results. Key statistics of these water quality parameters measured in this study are listed in Table 2.

### 2.3. Data Processing and Statistical Analyses

Prior to statistical analyses, the missing values were filled with the mean values of each quarter sequence. Then, logarithmic transformations were performed to standardize the environment variables so that the data could meet the assumptions of normality and homoscedasticity for statistical analyses (except for pH). Logarithmic transformation is a kind of standardization which can eliminate the influence of different units and scales of the measurements, make the data dimensionless and tends to reduce the effect of outliers [15,36]. The logarithmic transformation equation can be expressed as:

S_ij_ = Log_10_(x_ij_ + 1), I = 1, …, 12, j = 1, …, 45, (2)

where x_ij_ is the i-th variable of the j-th sample; S_ij_ is the standardized value of x_ij_. Whether the data obeyed a normal distribution could be determined by the standardized skewness and standardized kurtosis. The environmental variables, whose values of standardized skewness and standardized kurtosis were outside the range of −2 to +2, were considered to correspond to a non-normal distribution [23]. With this method, only 12 parameters with proper distribution were selected although more than 20 water environmental parameters were available. For example, chemical oxygen demand (COD) was not chosen for further analyses due to its low confidence in the log-normal distribution test.

Spearman correlations (a statistical test) were employed to reveal the relationships among the water quality parameters. A correlation coefficient near −1 or 1 means a strong negative or positive linear relationship between two variables, while one closes to 0 demonstrates a weak relationship. In this study, the relationships with correlation coefficients greater than 0.5 at the significant level of *p* < 0.01 were chosen for further detailed analysis.

Cluster analysis (CA) is a multivariate statistical technique that can investigate the similarities and dissimilarities between sampling sites, which groups the relevant objects into different aggregations based on their interdependent variables or characteristics [29,37]. Hierarchical agglomerative cluster analysis (HACA), the most common approach used in CA, was chosen to provide intuitive similarity relationships between temporal and spatial patterns [2,23]. HACA analysis was employed on the standardized dataset by the Ward’s method using squared Euclidean distances without any prior knowledge of the number of clusters [22,23]. The cases in each group clustered by this method have the smallest sum of squares deviation, and the clustering processes are presented in a dendrogram. The categories were based on the linkage distance D_link_/D_max_ × 100, where D_link_ is the distance between two clusters or objects, and D_max_ is the maximum D_link_.

One-way analysis of variance (ANOVA) was performed to compare the differences of water quality variables in different seasons or sampling sites (*p* < 0.05; least-significance difference, LSD of assuming homogeneity of variance; Tamhane’s T2 of non-homogeneity of variance). ANOVA results were employed to show the reliability of CA results.

Principal component analysis (PCA) is another multivariate statistical technique that can reduce dimensionality of the original data set to extract the information about correlation among variables analyzed in the water samples with a minimum loss of the original information [30,38]. PCA was carried out to explore the main parameters that can explain the data variance in the groups divided by CA. The Kaiser-Meyer-Olkin (KMO) and Barlett’s tests were performed to test the suitability of the data. All the data calculations and statistical analyses in this study were performed by IBM SPSS 19 (SPSS Inc., Chicago, IL, USA) and Statistica 10 (Statsoft Inc, Tulsa, OK, USA).

## 3. Results and Discussion

### 3.1. Physicochemical Characteristics and Correlation Analyses

**Figure 2:**
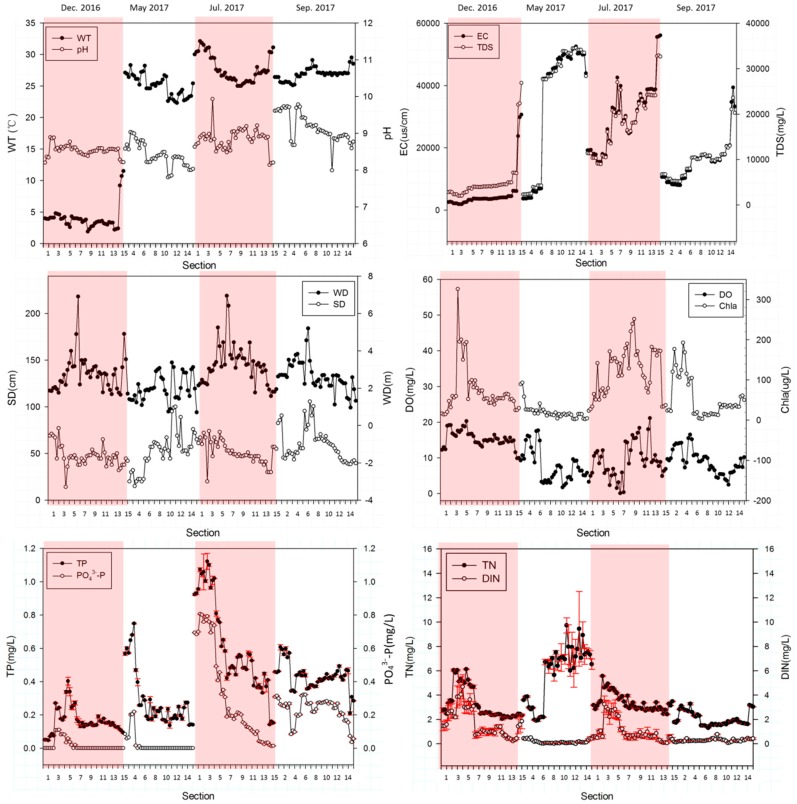
Spatial and temporal variability of water quality in the Duliujian River. Note: The major ticks of the *x*-axis are the third site at each section, and the minor ticks before each major ticks are the first and second site. i.e., the major tick number 1 means S1.3, two minor ticks before number 1 means S1.1, S1.2. The major tick labels are also corresponding to their section number.

In order to analyze the spatial variations and the correlations of these water quality parameters monitored in this research, the values of these 12 water quality parameters were plotted in Figure 2 corresponding to four different seasons. The Spearman correlation coefficients between these 12 physicochemical parameters were also calculated and are shown in Table 3.

As can be seen in Figure 2, WT, with a range of 1.9 °C to 32.1 °C across the study period, varied due to the season. It was also noticeable that WT had significant positive correlations with EC and TDS, which may rely on the enhancement of irons activity due to increasing WT. In addition, seawater intrusion due to the opening of estuarine sluices in summer and autumn (with higher WT) may also be responsible to the much higher EC (with an annual value of 20841.1 μs/cm) and TDS (with an annual value of 13,765.4 mg/L) than that of fresh water (TDS of drinking water is no more than 1000 mg/L [39]). As such, the correlations of WT with EC and TDS observed in this research were different from some other studies due to the effects of seawater intrusion [31,40].

With an average of 8.7, pH varied from 7.8 to 9.9 and indicated the slightly alkaline characteristic of water. The effects of pH on PO_4_^3−^-P adsorption should be responsible for strong positive correlations between TP and PO_4_^3−^-P. Under alkaline conditions, phosphorus was mainly released by ion exchange, by which, metal cations combined with phosphate are replaced by OH^−^ therefore leading to a higher dissolved PO_4_^3−^-P concentration [41,42]. Meanwhile, the negative charges on the surface sediment increased gradually with the increase of pH value [20], and the increased repulsion between the more negatively charged phosphate species and negatively charged surface sediment resulted in the lower adsorption of PO_4_^3−^-P [43]. PO_4_^3−^-P, therefore, was released into water and raised the TP concentration. The correlations between pH and TN, DIN (Table 3) indicated that the increase of pH might have a negative impact on biochemical processes related to dissolved organic nitrogen (DON) such as phytoplankton secretion, nitrogen fixation and absorption of aquatic plants [5,6,44].

The relationship between EC and TDS is well known and they had similar spatial trends in this study as the values increased along the river with significant increases at the last three sites in summer, autumn and winter, while a sharp growth from S6.3 (EC: 6940.0 μs/cm, TDS: 4251.0 mg/L) to S7.1 (EC: 42,128.0 μs/cm, TDS: 27,618.5 mg/L) was seen in spring. Besides, the correlation between DIN and EC might be attributed to microbial activity.

SD and WD also had similar spatial variation tendencies except for spring when a rubber dam under construction led to artificial effects on WD (S6.3, S7.1). The minimum values of SD and WD were 14.2 cm (winter, S4.1) and 0.7 m (spring, S15.3), respectively. Specifically, there were no strong correlations between SD/WD and other factors.

DO, an important index in water quality, exhibited a higher concentration in winter and a lower concentration in summer with a minimum value of 0.05 mg/L at S7.1. The physical processes of water should be responsible to the moderate negative correlations between DO and EC, TDS. Furthermore, the changes of aquatic plants and plankton and therefore their production ability of oxygen may also be another important factor influencing the DO concentration.

Chla displayed a large temporal variation with a lowest concentration of 2.2 μg/L at S13.2 in spring. As an important index representing the phytoplankton biomass, Chla did not exhibit a significant correlation with TN indicating that phytoplankton were affected by other limiting environmental factors rather than TN, which required more further study.

TP and PO_4_^3−^-P showed significant downtrend along the river across the seasons, varying from 0.05 to 1.12 mg/L and 0.00 to 0.81 mg/L, respectively. TP and PO_4_^3−^-P were associated well with WT, which indicated that these correlations might be caused by sediment release or aquatic life absorption, or the frequent anthropogenic activities such as agricultural land fertilized or aquaculture waste water discharge in spring and summer.

TN displayed the highest concentrations (9.7 mg/L) at S11.1 in spring and lowest concentration (1.3 mg/L) at S7.2 in autumn. DIN exhibited almost same variation trends with TN in four seasons, varying from 0.00 to 4.68 mg/L with an average of 0.83 mg/L.

### 3.2. Spatial Sites Grouping and Source Identification

**Figure 3:**
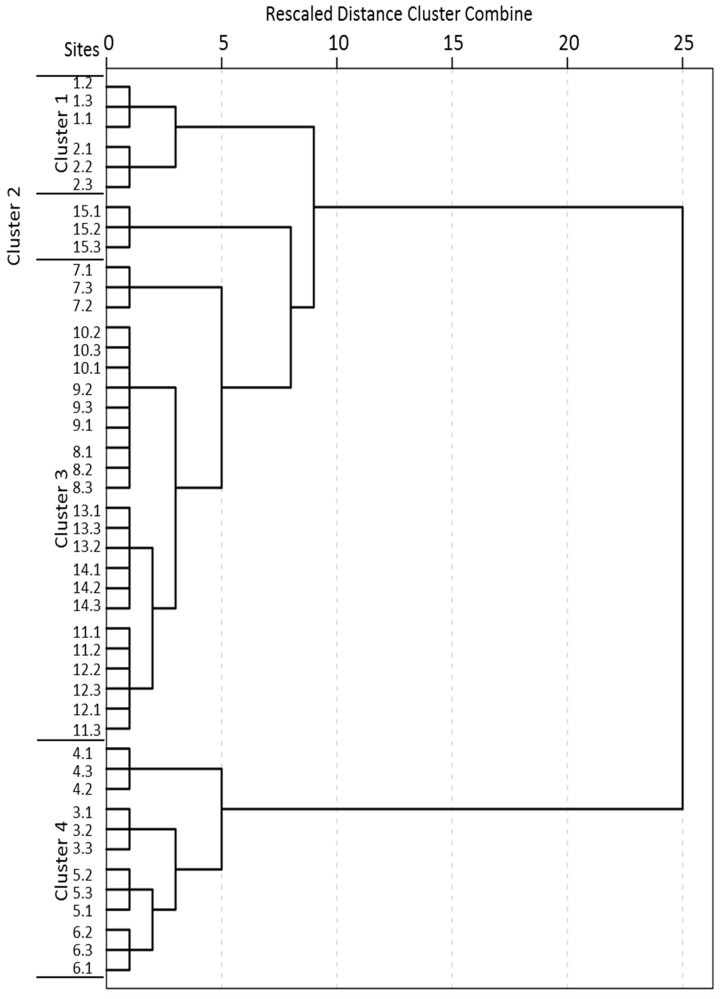
Dendrogram showing clustering of sampling sites according to Ward’s method using squared Euclidean distance.

**Figure 4:**
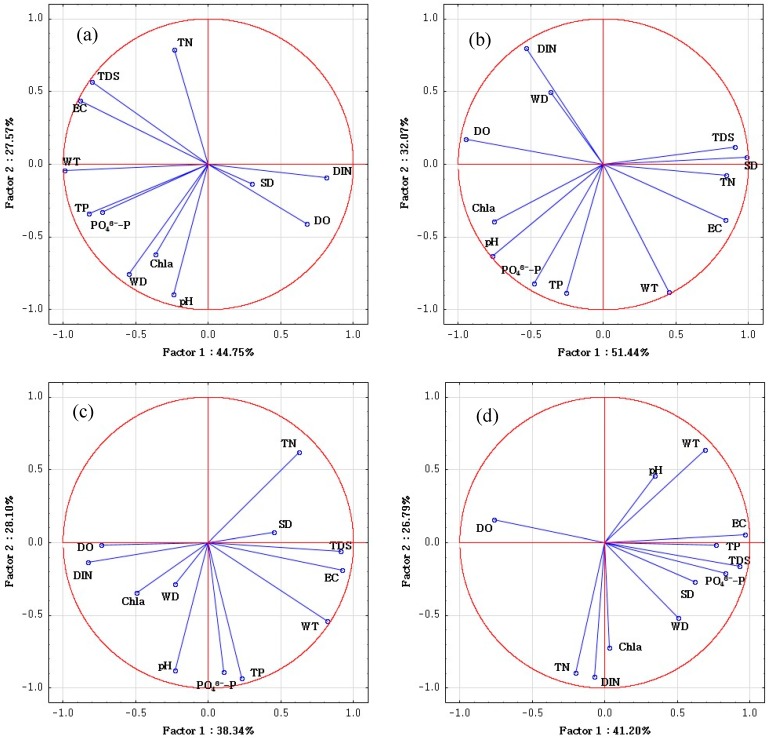
(**a**) PCA of cluster 1 of spatial CA; (**b**) PCA of cluster 2 of spatial CA; (**c**) PCA of cluster 3 of CA; (**d**) PCA of cluster 4 of spatial CA.

CA was used to categorize the spatial similarity groups of all the monitored sites according to their water quality similarities. EC and PO_4_^3−^-P, highly correlated with TDS and TP, respectively, were not used in CA to avoid repeated counting of the contribution rates of similar water quality parameters. As can be seen in Figure 3, the 45 sampling sites were categorized into four clusters at (D_link_/D_max_) × 100 < 32, and these sampling sites showed a reasonable consistency in their cross sections. Besides, ANOVA was performed to demonstrate the significance and reliability of the grouping result (Table 4). It was illustrated that the results of CA were effective for the significant differences of each water quality parameter among each cluster at *p*-value < 0.05 level. As shown in Table 5 and Figure 4, PCA was employed to find out the potential factors corresponding to these four clusters. In Table 5, it can be seen that four, two, four and four principal components for the water quality parameters were extracted in cluster 1, cluster 2, cluster 3 and cluster 4, respectively. In order to identify the main pollution source of each river reach, the spatial CA and PCA results can be comprehensively analyzed.

These clusters were determined by judging their water quality, which was primarily influenced by land use. Sites within each cluster have similar features and pollution source types. Cluster 1 contained S1.1–S2.3 (S1–S2) which were located in the upstream part of the Duliujian River. A large number of villages and fewer factories in this area indicated that these sampling sites were polluted by domestic waste water and agricultural activities. Cluster 1 was perceived as a relatively low pollution region because, as Figure 2 shows, the concentrations of nutrient like TN, TP of the S1.1–S2.3 were relatively lower in the sampling sites, especially in comparison with the following three sections. PCA shows that PC1 including WT, DO, EC, TDS, WD, TP, PO_4_^3−^-P and DIN, which explained 44.75% of the total variance, was the dominant component and attributed to the soil erosion, domestic sewage and agricultural activities. Soil erosion may increase the ion concentration leading to a significant effect on DO, EC and TDS. The high loadings of TP, PO_4_^3−^-P and DIN might be attributed to domestic sewage and surface runoff from farmland containing a large amount of nutrients [45]. PC2 explaining 27.57% of the total variance was attributed to the physicochemical indexes related to phytoplankton growth for the moderate and high loadings of TDS, pH, WD, TN and Chla.

Cluster 2 contained S15.1–S15.3 (S15) located on the last cross-section at the estuary. It mainly contributed to the exceptional EC and TDS due to the sea water intrusion (PC1, 51.44%) [17]. Because of the groundwater overpumping, land subsidence and natural geological conditions, the hydrodynamic balance between seawater and fresh water was destroyed and resulted in sea water intrusion. River and soil were therefore degenerated as a result of serious sea water intrusion.

Cluster 3 included S7.1–S14.3 (S7–S14) located on the middle and lower reaches of the investigated river. PC1, which accounts for 38.34% of the total variance, was attributed to the frequent intrusions of effluents from fisheries and livestock activities and mixing of seawater and river water. Due to the thriving fisheries [23], excessive fish feed and large amounts of fish waste containing a large number of nutrients were directly dumped into the river. As for livestock, sewage from washing farming sites and untreated poultry manure and urine were dissolved in rainfall runoff and then discharged into the river. Hence, DIN, as the dominant component of fish feed and poultry manure, was noticeable in PC1. Besides, from the textile, pharmaceutical, petroleum chemical and oil refinery factories, etc. along the river reach, a large number of industrial effluents containing acidic pollutants and phosphorus were discharged into the river, and became significant point contamination sources. As the PC2 shows, 28.10% of the total variance was explained by this component with moderate and high loadings of WT, pH, TN, TP and PO_4_^3−^-P. Although there are a lot of point and non-point source pollutions, this reach was grouped as moderate pollution, this must significantly rely on that all these sampling sites were located in the Beidagang wetland nature reserve where the self-purification ability and assimilative capacity were reasonably strong.

Cluster 4 comprising S3.1–S6.3 (S3–S6) was perceived as highly polluted because of the high nutrient concentration (PC1, 41.20% and PC2, 26.79%). In this region, there are many small factories producing feed, furniture and pharmaceuticals. High loading values of TP and PO_4_^3−^-P in this reach should be attributed to the effluents from feed mills which contained a lot of nutrient, and high loading values of DO was attributed to the effluents from furniture and pharmaceutical factories containing rich organic substance (as seen in Table 6, high emission of COD), which may consume a large amount of oxygen and resulted in low-oxygen environment [1] and therefore were the mainly point pollution sources in this region. The relatively intensive residential population surrounding the up-middle reaches of the investigated river also made that the domestic wastewater became an important point pollution source. Besides, livestock activities were a notable contributor to the non-point pollution sources since there are many poultry farms. PC2 with high loading values of TN, Chla and DIN was attributed to point pollution sources and nutrients related to phytoplankton. As shown in Table 6, this cluster has a high emission of NH_4_^+^-N which increased the TN and provided important nutrients for phytoplankton growth.

### 3.3. Temporal Periodic Grouping and Source Identification

**Figure 5:**
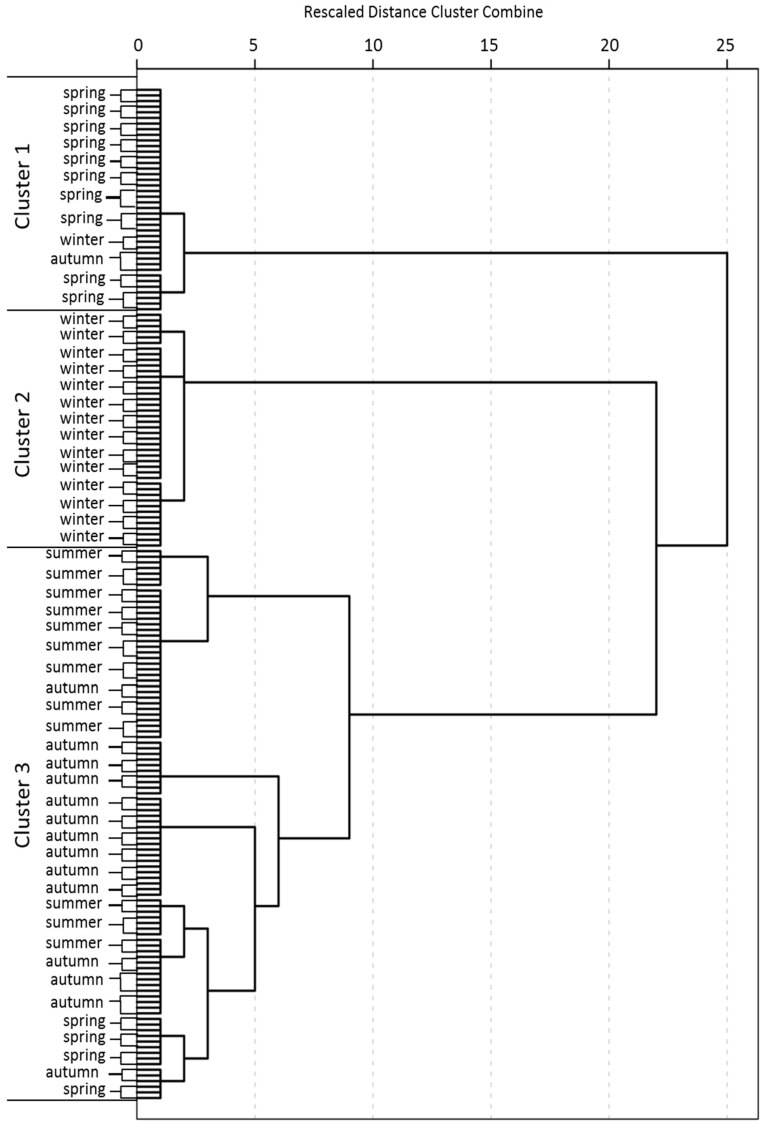
Dendrogram showing clustering of seasons according to Ward’s method using squared Euclidean distance.

The temporal similarity grouping results are shown in Figure 5. The 45 sampling sites in four seasons were grouped into three clusters at (D_link_/D_max_) × 100 < 60. EC and PO_4_^3−^-P were not includedin CA for the same reason as 3.2. The ANOVA was performed to demonstrate the significance and reliability of the grouping result (Table 7). All parameters contributed to the CA results since the differences of these water quality parameters among three clusters were significant at the *p*-value < 0.05 level. Hence, the grouping results of CA were proved to be effective. It was noteworthy that the temporal samples were grouped into three different clusters rather than four (seasons). These clusters can be explained by the hydrological characteristics of mean, high and low flow periods which were divided by the mean precipitation in 1980–2010 [46], consistent with [28]. In the Duliujian River watershed, spring and autumn with the seasonal precipitations of 0.17 × 10^8^ m^3^, 0.21 × 10^8^ m^3^ were classified as mean flow period, while summer (0.82 × 10^8^ m^3^) and winter (0.03 × 10^8^ m^3^) were the high and low flow periods, respectively. Sampling data, anthropogenic activities and natural conditions may provide some explanations for the grouping results.

**Figure 6:**
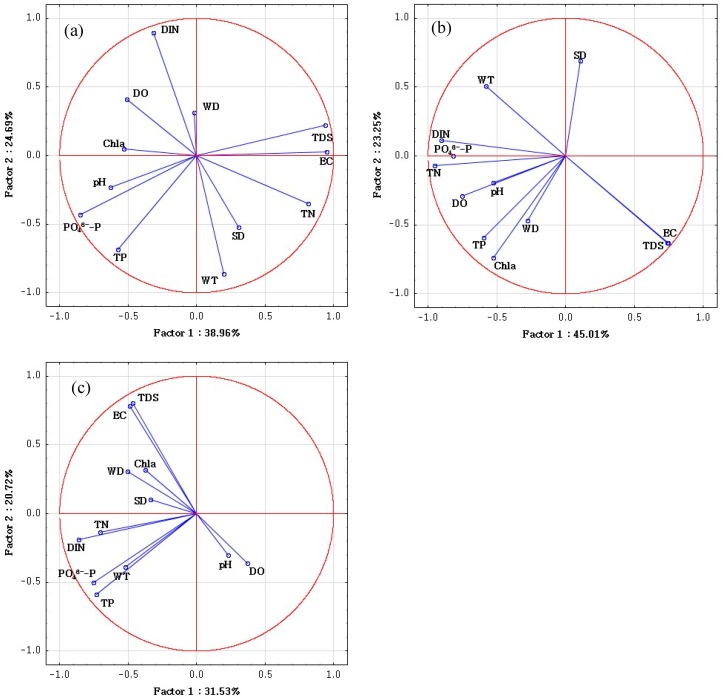
(**a**) PCA of cluster 1 of temporal CA; (**b**) PCA of cluster 2 of temporal CA; (**c**) PCA of cluster 3 of temporal CA.

In addition, PCA was employed to find out the main factors for these three clusters. PCA extracted four, three and four principal components for the water quality parameters in the mean, high and low flow periods, respectively. Further, these PCs with eigenvalues larger than 1 explained 82.40%, 79.13% and 82.49% of the total variance according to the relevant data sets (Table 8, Figure 6). All the significances of Bartlett’s sphericity test were below 0.001 and the corresponding results of Kaiser-Meyer-Olkin (KMO) were 0.53, 0.60 and 0.51, which indicated the reliability of the PCA results. In this study, the loadings were classified as ‘strong’, ‘moderate’, and ‘weak’ according to the loading values of >0.70, 0.70–0.50, and 0.50–0.30, respectively [47,48].

In detail, Cluster 1 consisted of almost all the sampling sites in May 2017 which corresponding to the mean flow period. Except that, some of the results that not divided into this cluster were mainly affected by the construction of water conservancy project. During the sampling period, a rubber dam was being built between the cross S6 (S6.1, S6.2 and S6.3) and S7 (S7.1, S7.2 and S7.3). Hence, the samplings S3.1–S6.3 were taken from a lake-like area with no water supply and discharge, which resulted in the fact that the S3.1–S6.3 with relatively lower EC, TDS and higher nutrient were grouped into Cluster 2. Besides, S7.1–S15.3 showed higher EC and TDS due to the seawater intrusion in comparison with the other three seasons. In PCA results for mean flow period (corresponding to Cluster 1), the first principal component (PC1) explained 38.96% of the total variance and exhibited strong positive loadings on EC, TDS and TN, while a strong negative loading on PO_4_^3−^-P and moderate negative loadings on DO, pH, TP and Chla. PC1 was mainly attributed to natural processes and nutrient pollution. Seawater intrusion brought a large number of ions such as Na+, Mg^2+^, Ca^+^ as well as Cl^−^ into the river, which significantly increased the values of EC and TDS [17]. The positive loading with pH was attributed to the seawater intrusion exacerbated the soil erosion, which improved the dissolved CO_3_^2−^ and HCO^3−^ from rock dissolution [28,40]. Besides, nutrients like TN, TP, and PO_4_^3−^-P were mainly from domestic wastewater, agricultural and livestock activities [20]. PC2, which explained 24.69% of the total variance, had high negative loadings on WT and moderate negative loadings on SD and TN DIN, while high positive loadings on DIN. This component may represent the metabolism of microorganisms since DIN was associated with nitrification and denitrification closely [7]. PC3, accounting for 11.66% of the total variance showed moderate negative loading on DO and positive loading on SD. This component was regarded as the oxidation process since the effluents from furniture factories and pharmaceutical factories contained a lot of organic substance, whose oxidation process consumed a large amount of DO, and reduce the SD. PC4 was interpreted as the physical process component and may be associated with the seasonal effect of the flow.

Cluster 2 consisted of almost all the sampling sites in December 2016, which corresponds to the low flow period. In this cluster, DO was relatively high and the content of TN and TP were moderate. PC1 for this cluster had strong positive loadings on EC and TDS, while strong negative loadings on DO, TN, PO_4_^3−^-P, and DIN and moderate loadings on WT, pH, TP and Chla. This component was attributed to natural processes (seawater intrusion), metabolic process of microorganisms (anaerobic fermentation processes [36], nitrification and denitrification) and anthropogenic activities (effluents from factories, wastewater from livestock and domestic). PC2 had strong loading on Chla, moderate loadings on WT, EC, TDS SD, and TP. This component was considered as phytoplankton growth processes. Compared to the high flow period, EC and TDS became important factors, and TP became the limited factor for phytoplankton. PC3, explaining 10.87% of the total variance with the negative loadings on DO and pH, representing the oxidation of wastewater.

Cluster 3 included almost all the sampling sites in July 2017 and September 2017 corresponding to the high flow period. It can be seen from the PCA results for this cluster (corresponding to high flow period), PC1 accounting for 31.53% of the total variance, had strong negative loadings on TN, TP, PO_4_^3−^-P and DIN, moderate loadings on WT and WD. This component was interpreted as due to anthropogenic processes [19]. Effluents from factories, waste water from domestic, fisheries and livestock activities containing a lot of nutrients were probably the mainly reason. In this period, the average NH_4_^+^-N and TP emission concentrations of the 24 swage outlets along the river were 2.49 mg/L and 0.47 mg/L according to the data from Tianjin Eco-environmental monitoring center, which demonstrated high nutrients input. PC2 had strong positive loadings on EC, TDS, while moderate negative loadings on TP and PO_4_^3−^-P, representing the natural processes such as seawater intrusion and sediment absorption. PC3 explaining 16.40% of the total variance, had strong loadings on SD and Chla, moderate loading on TN, reflecting the phytoplankton growth process. Chl a was an index represented phytoplankton biomass, and SD and TN were the important factors for plankton growth [36]. PC4, accounted for 13.84% of the total variance, had strong positive loading on pH, moderate loadings on WT, DO and WD, reflecting the oxidation processes. Effluents from feed mills, petrochemical and pharmaceutical factories may contain carbohydrates which hydrolyze to produce acids or organic acids, thus decreasing the pH values.

Overall, PCA indicated the temporal variations of water pollutants. TN, TP and PO_4_^3−^-P, were always in PC1 which indicated that nitrogen and phosphorus were the main basic factors in all periods, should therefore more attention be paid to nutrient control and effluents from sewage outlets. For the high flow period, microbes and phytoplankton metabolized and reproduced quickly with the high nutrient concentration. According to the algal measurements in the study period, the density of *Microcystis*, which can reflect the condition of algal bloom, was much higher in summer (3.69 × 10^6^ ind./L) and autumn (57.79 × 10^6^ ind./L) than the spring (0.03 × 10^6^ ind./L) and winter (0.17 × 10^6^ ind./L). More attention should be paid to the potential risk of algal blooms. Significant loadings of EC and TDS in PC1 except for the high flow period indicated that a natural process (seawater intrusion) was the main basic factors influencing water quality in mean and low flow periods, and large flows can reduce the impacts of seawater intrusion.

## 4. Conclusions

In this study, the spatial and temporal variations in water quality of Duliujian River were identified using statistical techniques (Spearman correlation, CA, ANOVA and PCA). This study revealed that the water quality parameters varied reasonably with season and spatial position.

The results of spatial CA and PCA revealed that the most polluted reach was the up-middle reaches, including S3.1–S6.3. This reach was mainly polluted by nutrients from feed mills, furniture and pharmaceutical factories as well as livestock activities. The middle and lower reaches of the river, S7.1–S14.3, were moderately polluted. Though it was contaminated by complex pollutants from up-middle reaches as well as surrounding factories, it showed good self-purification ability since the river crosses some wetlands. The upstream of the river including S1.1–S2.3 was lightly polluted, since it was only polluted by domestic wastewater and agricultural activities. The estuary of the river, S15.1–S15.3, was influenced significantly by seawater intrusion.

The results of temporal CA showed that the temporal samples were grouped according to their hydrological characteristics into mean, low and high flow periods rather than the four seasons. Temporal PCA indicated that nitrogen and phosphorus were mainly the basic factors in all periods, and nutrient control and effluents from sewage outlets should therefore be paid more attention. Specially to the high flow period, more attention should be paid to the potential risk of algal blooms since that phytoplankton metabolized nutrients and reproduced quickly with high concentration nutrient. Significant loadings of EC and TDS in PC1 indicated that seawater intrusion was the mainly basic factors influencing water quality in mean and low flow periods.

Proper treatment for domestic and livestock wastes as well as polluted surface runoff should be undertaken to reduce the nutrient concentration. Management needs to be carried out to minimize effluent pollution from factories. These results can help managers gain insights into the temporal and spatial variations in water quality of a seagoing river ecosystem and consequently make better ecological management decisions.

## Figures and Tables

**Table 1:** The water quality parameters and their abbreviation, analytical method and units.

Parameters	Abbreviation	Analytical Method	Unit
Water temperature	WT	YSI ProPlus	°C
Water transparency	SD	Secchi disk	cm
Water depth	WD	Meter stick	m
pH	pH	YSI ProPlus	
Dissolved oxygen	DO	YSI ProPlus	mg/L
Electrical conductivity	EC	YSI ProPlus	μs/cm
Total dissolved solids	TDS	YSI ProPlus	mg/L
Total nitrogen	TN	Alkaline potassium persulfate digestion UV spectrophotometric method	mg/L
Total phosphorus	TP	Ammonium molybdate spectrophotometric method	mg/L
Ammonia nitrogen	NH_4_^+^-N	Nessler’s reagent spectrophotometric method	mg/L
Nitrate	NO_3_^−^-N	Gas phase molecular Absorption spectrum method	mg/L
Nitrite	NO_2_^−^-N	Gas phase molecular Absorption spectrum method	mg/L
Orthophosphate	PO_4_^3−^-P	molybdenum-antimony anti-spectrophotometric method	mg/L
Chlorophyll a	Chla	spectrophotometric method	μg/L
Dissolved inorganic nitrogen	DIN	NH_4_^+^-N + NO_3_^−^-N + NO_2_^−^-N	mg/L

**Table 2:** Key statistics of the water quality parameters in the Duliujian River.

Parameters	*n*	Min.	Max.	Mean	S.D.	Median
WT (°C)	174	1.90	32.10	20.78	10.16	25.75
SD (cm)	174	14.20	105.50	52.76	16.63	50.00
WD (m)	174	0.70	6.94	2.68	1.02	2.66
pH	174	7.80	9.93	8.74	0.42	8.69
DO (mg/L)	174	0.05	21.10	10.38	4.90	9.86
EC (μs/cm)	174	1900.00	56,066.00	20,841.12	16,582.06	17,039.50
TDS (mg/L)	174	2008.50	34,515.00	13,765.38	10,449.19	10,374.00
TN (mg/L)	174	1.30	9.73	3.57	1.91	2.90
TP (mg/L)	174	0.05	1.12	0.38	0.24	0.36
PO_4_^3−^-P (mg/L)	174	0.00	0.81	0.15	0.20	0.08
Chla (μg/L)	174	2.22	326.01	68.92	62.34	50.45
DIN (mg/L)	174	0.00	4.68	0.83	1.00	0.43

**Table 3:** Correlation analysis of water quality parameters (Spearman correlation coefficients(r)).

Parameters	WT	DO	EC	TDS	pH	SD	WD	TN	TP	PO_4_^3−^-P	Chla	DIN
WT	1											
DO	**−0.38 ^a^**	1										
EC	**0.35 ^a^**	**−0.69 ^a^**	1									
TDS	**0.26 ^a^**	**−0.68 ^a^**	**0.99 ^a^**	1								
pH	**0.48 ^a^**	**0.32 ^a^**	**−0.26 ^a^**	**−0.30 ^a^**	1							
SD	0.16 ^b^	**−0.41 ^a^**	**0.25 ^a^**	**0.27 ^a^**	−0.04	1						
WD	−0.06	0.13	−0.06	−0.02	**0.25 ^a^**	0.10	1					
TN	**−0.19 ^a^**	−0.18 ^b^	**0.39 ^a^**	**0.38 ^a^**	**−0.55 ^a^**	0.09	0.02	1				
TP	**0.68 ^a^**	−0.17 ^b^	0.10	0.02	**0.65 ^a^**	0.03	**0.23 ^a^**	−0.12	1			
PO_4_^3−^-P	**0.69 ^a^**	−0.19 ^b^	−0.01	−0.06	**0.69 ^a^**	**0.25 ^a^**	**0.28 ^a^**	**−0.28 ^a^**	**0.86 ^a^**	1		
Chla	−0.03	**0.39 ^a^**	**−0.28 ^a^**	**−0.29 ^a^**	**0.33 ^a^**	**−0.47 ^a^**	**0.45 ^a^**	−0.01	**0.35 ^a^**	**0.24 ^a^**	1	
DIN	−0.18 ^b^	**0.46 ^a^**	**−0.51 ^a^**	**−0.48 ^a^**	0.08	−0.10	**0.33 ^a^**	−0.00	0.02	0.15 ^b^	**0.47 ^a^**	1

Note: Bold, the most significant values at 0.01 level. ^a^ correlation is significant at the 0.01 level (2-tailed). ^b^ correlation is significant at the 0.05 level (2-tailed). Shading background, correlation coefficient is greater than 0.5 at the level of 0.01.

**Table 4:** The results of ANOVA of spatial CA.

	WT	DO	EC	TDS	pH	SD	WD	TN	TP	PO_4_^3−^-P	Chla	DIN
df	44	44	44	44	44	44	44	44	44	44	44	44
F-statistics	54.27	8.07	152.18	142.51	40.30	4.73	4.49	4.65	88.07	73.35	28.94	61.10
*p*-value	<0.001	<0.001	<0.001	<0.001	<0.001	0.006	0.008	0.007	<0.001	<0.001	<0.001	<0.001

Note: df is the degree of freedom.

**Table 5:** Component loadings of each parameters and cumulative variance of the principal components.

**C1**	**WT**	**DO**	**EC**	**TDS**	**pH**	**SD**	**WD**	**TN**	**TP**	**PO_4_^3−^-P**	**Chla**	**DIN**	**Eige.**	**Cum.%**
PC1	**−0.99**	**0.68**	**−0.88**	**−0.80**	−0.24	0.30	**−0.55**	−0.23	**−0.82**	**−0.73**	−0.36	**0.81**	5.37	44.75
PC2	−0.04	−0.41	0.44	**0.57**	**−0.90**	−0.14	**−0.76**	**0.79**	−0.34	−0.33	**−0.62**	−0.09	3.31	72.32
PC3	0.06	−0.46	0.04	0.04	−0.01	**0.88**	0.17	**−0.53**	−0.06	0.03	**−0.67**	−0.16	1.78	87.19
PC4	−0.07	−0.21	−0.11	−0.13	−0.31	0.01	−0.26	−0.07	0.45	**0.59**	0.00	**0.52**	1.07	**96.11**
**C2**	**WT**	**DO**	**EC**	**TDS**	**pH**	**SD**	**WD**	**TN**	**TP**	**PO_4_^3−^-P**	**Chla**	**DIN**	**Eige.**	**Cum.%**
PC1	0.45	**−0.95**	**0.84**	**0.90**	**−0.76**	**0.99**	−0.36	**0.85**	−0.26	−0.48	**−0.75**	**−0.53**	6.17	51.44
PC2	**−0.88**	0.17	−0.38	0.12	**−0.63**	0.05	**0.50**	−0.08	**−0.88**	**−0.82**	−0.39	**0.80**	3.85	**83.52**
**C3**	**WT**	**DO**	**EC**	**TDS**	**pH**	**SD**	**WD**	**TN**	**TP**	**PO_4_^3−^-P**	**Chla**	**DIN**	**Eige.**	**Cum.%**
PC1	**0.82**	**−0.74**	**0.92**	**0.91**	−0.23	0.45	−0.23	**0.62**	0.23	0.10	−0.49	**−0.83**	4.60	38.34
PC2	**−0.54**	−0.01	−0.19	−0.06	**−0.88**	0.07	−0.28	**0.62**	**−0.93**	**−0.89**	−0.34	−0.14	3.37	66.44
PC3	0.05	0.14	0.29	0.36	−0.07	**−0.67**	0.26	0.34	0.14	−0.37	**0.69**	0.06	1.51	79.02
PC4	0.03	0.11	−0.01	−0.03	0.11	−0.47	**−0.85**	−0.21	0.03	−0.02	−0.03	−0.28	1.08	**88.05**
**C4**	**WT**	**DO**	**EC**	**TDS**	**pH**	**SD**	**WD**	**TN**	**TP**	**PO_4_^3−^-P**	**Chla**	**DIN**	**Eige.**	**Cum.%**
PC1	**0.69**	**−0.76**	**0.97**	**0.93**	0.35	**0.62**	**0.51**	−0.20	**0.76**	**0.83**	0.03	−0.07	4.94	41.20
PC2	**0.63**	0.16	0.06	−0.16	0.46	−0.27	**−0.52**	**−0.89**	−0.02	−0.21	**−0.72**	**−0.92**	3.22	67.99
PC3	0.25	−0.27	−0.02	−0.12	−0.44	**−0.52**	**−0.54**	0.28	0.49	0.24	−0.12	0.14	1.33	79.06
PC4	−0.06	0.49	−0.17	−0.19	**0.59**	0.13	−0.14	0.19	0.35	0.41	−0.13	0.22	1.09	**88.14**

Note: CX means cluster X; Eige. means Eigen values; Cum. means Cumulative.

**Table 6:** Number of sewage draining outlets and annual emissions of NH4 and COD in each cluster.

Cluster Number	Number of Sewage Draining Outlets	NH_4_ (t/a)	COD (t/a)
1	2	33.1	250.07
2	0	0.00	0.00
3	6	157.25	1308.94
4	17	369.18	3458.81

**Table 7:** The results of ANOVA of temporal CA.

	WT	DO	EC	TDS	pH	SD	WD	TN	TP	PO_4_^3−^-P	Chla	DIN
df	179	179	179	179	179	179	179	179	179	179	179	179
F-statistics	2671.44	48.16	263.96	123.02	133.02	5.81	7.23	80.90	127.59	61.97	40.99	48.39
*p*-value	<0.001	<0.001	<0.001	<0.001	<0.001	0.004	0.001	<0.001	<0.001	<0.001	<0.001	<0.001

Note: df is the degree of freedom.

**Table 8:** Component loadings of each parameters and cumulative variance of the principal components.

**MFP**	**WT**	**DO**	**EC**	**TDS**	**pH**	**SD**	**WD**	**TN**	**TP**	**PO_4_^3−^-P**	**Chla**	**DIN**	**Eige.**	**Cum.%**
PC1	0.20	**−0.51**	**0.95**	**0.94**	**−0.63**	0.30	−0.02	**0.82**	**−0.57**	**−0.85**	**−0.53**	−0.32	4.68	38.96
PC2	**−0.86**	0.41	0.03	0.22	−0.23	**−0.52**	0.31	−0.35	**−0.69**	−0.43	0.05	**0.89**	2.96	63.65
PC3	−0.22	**−0.69**	−0.18	−0.14	−0.41	**0.55**	0.34	−0.22	−0.07	0.04	0.41	0.09	1.40	75.31
PC4	0.17	−0.08	0.11	0.06	−0.30	−0.19	**−0.79**	−0.20	−0.11	−0.01	0.49	0.07	1.09	**84.40**
**LFP**	**WT**	**DO**	**EC**	**TDS**	**pH**	**SD**	**WD**	**TN**	**TP**	**PO_4_^3−^-P**	**Chla**	**DIN**	**Eige.**	**Cum.%**
PC1	**−0.58**	**−0.75**	**0.74**	**0.74**	**−0.52**	0.10	−0.28	**−0.96**	**−0.60**	**−0.82**	**−0.52**	**−0.91**	5.40	45.01
PC2	**0.51**	−0.29	**−0.63**	**−0.63**	−0.19	**0.69**	−0.47	−0.07	**−0.59**	0.00	**−0.74**	0.12	2.79	68.26
PC3	0.22	**−0.57**	−0.04	−0.05	**−0.79**	−0.17	0.36	0.13	0.14	0.27	0.16	0.01	1.30	**79.13**
**HFP**	**WT**	**DO**	**EC**	**TDS**	**pH**	**SD**	**WD**	**TN**	**TP**	**PO_4_^3−^-P**	**Chla**	**DIN**	**Eige.**	**Cum.%**
PC1	**−0.52**	0.37	−0.49	−0.47	0.23	−0.34	**−0.51**	**−0.71**	**−0.73**	**−0.75**	−0.38	**−0.86**	3.78	31.53
PC2	−0.39	−0.36	**0.78**	**0.80**	−0.30	0.10	0.31	−0.14	**−0.59**	**−0.50**	0.32	−0.19	2.49	52.26
PC3	−0.26	0.26	−0.14	−0.13	−0.28	**−0.80**	−0.12	**0.63**	0.11	−0.34	**0.73**	0.08	1.97	68.65
PC4	**−0.50**	0.45	−0.07	−0.04	**0.77**	0.31	**0.62**	0.02	0.04	0.04	0.37	0.02	1.66	**82.49**

Note: CX means cluster X; Eige. means Eigen values; Cum. means Cumulative.
